# Examining Meaningful Engagement Among Assisted Living Residents With Dementia

**DOI:** 10.1093/geroni/igaa057.2315

**Published:** 2020-12-16

**Authors:** Candace Kemp, Alexis Bender, Elisabeth Burgess, Joy Ciofi, Fayron Epps, Jennifer Morgan, Molly Perkins

**Affiliations:** 1 Georgia State University, Atlanta, Georgia, United States; 2 Emory University, Atlanta, Georgia, United States

## Abstract

Across care setting, lack of meaningful activity is identified by persons living with dementia and their care partners as a persistent and significant unmet care need. Here, we examine the opportunities for and experiences with meaningful engagement among assisted living residents with dementia using data from our 5-year NIA-funded qualitative study (R01AG062310). Guided by grounded theory method, the study involves in-depth interviews, participant observation, and resident record review collected over a one-year period in four diverse assisted living communities. Analysis of these data shows a range of engagement opportunities and experiences within and across care communities and point to the influence of resident (e.g. cognitive and physical function, interests, relationships), care partner (e.g., dementia knowledge, strategies, availability), and care community (e.g., size, location, resources) factors. We conclude by offering suggestions for policy and practice and for future research aimed at improving the quality of life of persons living with dementia.

